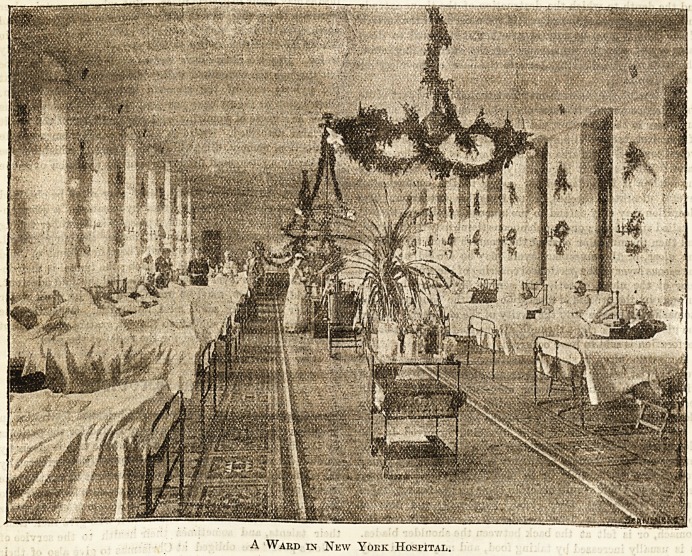# Extra Supplement.—The Nursing Mirror

**Published:** 1893-12-30

**Authors:** 


### "The Hospital" Nursing Mirror.

Being the Extra Nursing Supplement of "The Hospital" Newspaper.

[Contributions for this Supplement should be addressed to the Editor, The Hospital, 428, Strand, London, W.C., and should have the word "Nursing" plainly written in left-hand top corner of the envelope.]

### News from the Nursing World.

### ANOTHER PLEA FOR OUR SOLDIERS.

A good suggestion comes to us through the *Army and Navy Gazette.* Why should not the married women of battalions be trained to nurse the sick? "Enteric fever claims many victims in all grades of the army in India, but those who suffer most severely—and, it may be added, cost the country most—are the young men composing the rank and file." If such of the married women as are suited for the work could be trained to work under qualified superintendents, it would certainly be an excellent plan for the soldiers, and officers too. Moreover the objections to men marrying would assuredly be modified if their wives were found to be valuable certificated nurses. The suggestion appears one well worthy of consideration, and we should be glad to have the opinions of our readers on this important matter.

### THE THREE MONTHS' COURSE.

The three months' course seems gaining ground, and in some districts nursing certainly, as the Irishman said, appears to be "progressing backwards." There can be no objection to every woman in the country going in for three months' study or three months' ambulance work; but there is every reason why she should be restrained from afterwards passing herself off as a nurse. Even the pupils, often quite uneducated women, who are sent to institutions to learn midwifery in a few weeks, now return to isolated country districts to "take up" nursing. Monthly cases do not absorb all their time, so they are put on to attend to any and every kind of illness. The ladies in the neighbourhood utilise their services to save themselves the trouble and expense of getting a fully-trained nurse for a poor man's family; whilst the worried and over-worked country doctor has to take what he can get, although he may well sigh over the prevailing tendency amongst his well-to-do patrons to secure cheap, inefficient substitutes instead of fully-qualified district nurses for the sick poor. Adequate instruction in monthly nursing ought surely to be regarded as preparation for that branch of work only. It is misleading and dangerous to let loose on the public, under the title of nurses, women whose sole experience has been obtained from a three months' course.

### A WISE CHAIRMAN.

In tendering her resignation the other day, a nurse asked the Brentford Guardians to release her as soon as possible. The chairman pathetically inquired how it was that they could not keep a nurse for five minutes? and then answered his own question most pertinently by saying he was afraid nurses would not be persuaded to remain unless better accommodation was offered to them.

### UNSEEMLY LEVITY.

The Lowestoft Board of Guardians have engaged a permanent nurse. She was for three years at the Cambridge Infectious Hospital, but there is no mention of her having received any general training. There were two candidates for the vacant post, and some ill-timed jesting took place as to the favoured candidate having "captivated" the Guardians, "and none denied the soft impeachment." The sooner such uncalled-for comments are officially censured, the better will it be for the general position of Poor-law nurses.

### WORK FOR NURSES.

The number of patients suffering from infectious diseases in the district of Camborne has called for special action on the part of the Local Board, and they have concurred in the medical officer's judgment regarding the necessity for the immediate engagement of two trained nurses. The impossibility of the doctor's directions being carried out, either in the treatment of patients, or in preventing the spread of infection, is sufficiently obvious, for not only children, but their parents are in many cases laid up with scarlatina, influenza, &c., and therefore dependent on the kindness of their neighbours for the most ordinary attentions. Under such circumstances the commonest preventive measures must be in danger of neglect.

### A VALUED NURSE.

The Arbroath District Nursing Association, which is affiliated with the Q.V.J.I., has just issued its second annual report. There is an excellent record of work, and some of the cases quoted show most emphatically the necessity for district nurses being thoroughly trained and experienced. The Queen's nurse at Arbroath attended an abdominal operation case, a fractured skull, and a typhoid patient, who all made good recoveries, besides a great variety of other cases. It is pleasing to read at the end of the report that the committee granted to their nurse a special fortnight's holiday, in addition to the usual month, in consideration of the exceptional hard work of last winter. "The wisdom of this favour has been demonstrated by the renewed strength and unwearied devotion she has brought to bear on all her work since her return, and to this in great measure must be attributed the undoubted success which attends the association." There is every chance of success continuing to attend on a society managed in such wise and considerate fashion as the one at Arbroath.

### AMATEUR CLASSES.

With the fertility of expression which characterises them, the Americans have neatly suggested "amateur classes in nursing" as the suitable designation of a scheme for teaching ladies (and gentlemen too, if they like) some of the ordinary rules of health. It is proposed that a regular trained nurse, with experience of private nursing, should give instruction in simple family nursing, ventilation, &c. This course of instruction is to supplement the ordinary every-day usefulness of individuals. Pupils are to have the offer of a little knowledge, yet they are not to be tempted to believe that a handy amateur can supersede a trained nurse. The plan appears likely to find favour and to be practically developed by our American friends.

### AN EXPLANATION NEEDED.

"Inexplicable" is the appropriate heading of a letter to a Birmingham paper detailing the particulars of a transaction which sounds an extraordinary one. However, as most things, especially grievances, often admit of explanation, we may hope that the Secretary of the Queen's Hospital will show a good reason for a week's fees being demanded for the services of two nurses who were withdrawn from a case by the Matron after attendances of thirty-six hours and one night respectively. The husband of the patient, who gives his name and address, omits to state if an adequate reason was given by the Matron for the sudden recall of the nurses.

### UNATTENDED DEATH-BEDS.

Cordial thanks are due to the Rev. Father Sharp for calling attention to the terrible neglect of the patients at Skipton Workhouse during the night. In "Christian England" in the nineteenth century a poor woman was confined, was afterwards seized with convulsions, and ultimately died, with no one near to look after her, although an old and incapable woman was in the next room. The usual practice of ringing a bell to summon the day nurse was not followed in this instance. The latter has fourteen hours' day duty, and there is something truly inhuman in the custom of "ringing up" in the night a woman who has such long working hours. The revelations made by Father Sharp before the Skipton Board resulted in the engagement of a temporary night nurse being authorised, and it is to be hoped that a properly trained one will be secured.

### A SPECIAL MILK SUPPLY.

The difficulty of ensuring a regular supply of sterilised milk for babies and invalids, for whom it is prescribed, led some two years ago to the opening of a laboratory in Boston, where milk from selected cows is carefully treated. Physicians send their own prescriptions, and have milk modified to suit the requirements of their patients. The enterprise proving a scientific and financial success at Boston, a similar establishment has been opened in New York, and it is under discussion to start another in Philadelphia. This scheme should prove of practical benefit to delicate infants, who have unfortunately to be brought up by hand.

### IRELAND AND U.S.A.

A Training School Convention is to be held in New York in January to discuss many important questions bearing upon nursing subjects, such as length of service, and methods of instruction. A similar movement is afloat in Ireland, and we shall be interested to see the points of difference as well of resemblance between the cotemporaneous new departures.

### MELBOURNE.

The latest addition to the charitable institutions of Melbourne, the St. Vincent's Hospital, was formally opened on Monday, November 6th. The ceremony was performed by Lady Clarke, wife of Sir William Clarke, Bart., and the occasion was one of considerable display. A large number of prominent citizens of all denominations was present, together with the Archbishop, numbers of the clergy of the Roman Catholic Church, a few young men of the Church of England, and the Rev. Dr. Bevan, who was one of the speakers. The hospital has been purchased and equipped solely by donations, no Government aid being received, and will be nursed and administered by the Sisters of the Order of St. Vincent de Paul, whose convent adjoins the hospital. The out-patient department has been open for about a month, and patients are waiting to come in. A bazaar and an entertainment in aid of the funds will shortly be held.

### NURSES' SALARIES AT KIMBERLEY.

Readers interested in the prospects of nurses in South Africa will be glad of the following definite information, which has been sent to us from Kimberley Hospital Private Nursing Institution: "Staff nurses from other hospitals commence with salaries at £40 per annum, receiving £50 the second year; whilst nurses belonging to the Kimberley Hospital begin at £45 per annum. Private nurses are paid from £50 to £75. First-class passage out costs (with reductions) £35 10s. by ship, and £4 4s. by rail; second-class £21 14s. by ship, and £2 12s. 6d. by rail from Cape Town. That is to say, about £40 first, and £25 second-class altogether."

### WHERE ARE THE COUNCIL?

It is publicly stated that the Royal British Nurses' Association authorities are favourable to the coercion of probationers on the Sussex County Hospital principle. It is with regret that we record this decision, because the Association, like the Inquisition of former times, is apparently about to enter upon a crusade which may blight the prospects of many innocent young women who aspire to be nurses. Surely the choice of joining any association should be left to the nurse until experience and knowledge are gained. Instead of this, the Association, if the wiser heads on its Council do not promptly interfere, will be committed to the encouragement of tyranny at the hands of matrons, and to a policy which practically leaves the inexperienced nurse no alternative between loss of money and disfavour with the authorities, and joining an association to which she may prefer not to belong. Under these circumstances we would earnestly warn a would-be nurse not to enter an institution where such injustice prevails, but to remain free to decide for herself to join the R.B.N.A. or not, as in the light of her experience she may deem best. Further, we can but think that the committees of all well-conducted hospitals will be careful not to introduce the possibility of tyranny and coercion into their midst, by electing as their matron a member of any Association charged with a proselytising mission. The R.B.N.A. must endanger the future of both matrons and probationers unless its Council repudiates a policy of coercion, which few self-respecting women will submit to. This false move may yet be amended, and we cherish a hope that those members of the society who have recently joined, with the laudable intention of arresting steps injurious to the interests of the nursing body, will exercise their influence to prevent a step so discreditable to themselves and so fatal to all progress.

### SHORT ITEMS.

Newby Board of Guardians have appointed as nurse for their infirmary the only candidate who applied for the post, a Mrs. Newbold, who was trained at the Adelaide Hospital, Dublin, and worked for five years in the Lancaster Lunatic Asylum. After the appointment had been made it transpired that no proper quarters were set apart for the nurse, and a committee was selected to go into the matter.—The Hon. Mrs. C Chetwynd has given a subscription of £160 per annum to the charming Children's Hospital, Caldecote House Bushey Heath, where Mrs. Chetwynd's daughter has been for some time a probationer.—Lady Burns has given a donation to the Glasgow Training Home for Nurses which will enable the directors to complete and to furnish an additional house for the Nurses.—The diphtheria epidemic is abating at Hinckley.

### On the Nursing of Diseases of the Stomach.

### VII.—GASTRIC ULCER.

Ulcer of the stomach has been mentioned several times in considering the various symptoms that occur in diseases of the stomach. Its causes, symptoms, results, and dangers, as well as its treatment, must now be considered. But first must be clearly understood what is meant by gastric ulcer. Perhaps the most familiar form of ulceration is that of the leg. Anyone who has worked among the poor must have seen many cases of this. Such ulcers differ much in size and appearance, but all have in common loss of tissue, destruction of the skin over a certain area, and very commonly of the underlying structures as well. In describing any ulcer several points have to be noted, such as its position, its size, and the depth to which the destruction of the tissue extends and the appearance of the edge. If the ulcer is on the surface of the body it must be noticed whether it be fixed to deep parts or not, also the appearance of the surrounding skin. The edge may be shelving down to the floor of the ulcer or steep down to it.

An ulcer of the stomach may be situated in any part of its walls, but commonly it is near the pyloric end, and the smaller curvature in the posterior, not the anterior, wall. The size of the ulcer varies; it may be so small that a threepenny bit would cover it, or its area may be as large as the palm of the hand. When small it is generally round or oval, when large the outline is irregular, either owing to the confluence of several ulcerated patches, or to irregular extension. The ulceration may only extend to the mucous membrane, then it is called superficial; or the submucous tissue may be involved, and then the muscular coat of the stomach forms the floor of the ulcer, or the muscular layers may be eroded, and then the serous layer or the peritoneal covering of the stomach is the floor of the ulcer. Lastly, the serous layer may be eaten through by the process of ulceration, and the contents of the stomach may escape into the cavity of the abdomen. When the ulcer extends right through all the coats of the stomach perforation is said to have occurred.

A gastric ulcer is slow in healing. This is not to be wondered at, seeing that the presence of food in the stomach will act as an irritant, and the movements of the stomach recurring, prevent the rest so needful to the healing of an ulcer. There may be bleeding from the surface of an ulcer, or a blood vessel may be opened by being involved in the ulceration. Gastric ulcer seldom occurs before the age of ten or twelve. It most commonly occurs in girls or young women who are anæmic, or in men between forty and fifty years of age. The symptoms often come on gradually; there may be uneasiness after food, flatulency, and loss of appetite. Pain is usually referred to the pit of the stomach, or is felt at the back between the shoulder blades. It is usually increased by taking food, and may indeed only come on after a meal, and is of a burning or shooting character. Vomiting very commonly occurs after the pain, which may be greatly relieved by such an attack. Hæmatemesis frequently results from ulceration, and if a vessel has been eroded is apt to be profuse.

Gastric ulcers may heal, and the symptoms gradually disappear, and happily this is a frequent termination, but even after healing has begun, ulceration is apt to start afresh, and cause a relapse. The pain and vomiting attendant on taking food may lead to such debility that the patient sinks exhausted, or he may die from hæmatemesis, or perforation, collapse, or peritonitis.

In the treatment of gastric ulcer attention to diet is of the greatest importance, and is, in fact, the nurse's chief care. The stomach must be at rest as much as possible, and in some cases it may be undesirable to feed by the mouth at all, but to give nutrient enemata.

The least irritating food must always be given. Milk is the best diet; if this causes vomiting even when peptonised, meat juice can be tried. Nothing must be given hot, and warm tea or coffee is considered particularly harmful. Ice to suck may be grateful.

Great care must be exercised to change the diet gradually. Easily-digested food must be given for a considerable time.

Should sudden collapse occur the nurse must see that the heat of the body is maintained by hot bottles, &c., and summon medical aid as quickly as possible.

*Cancer of the Stomach.*—The symptoms of cancer of the stomach are somewhat similar to those of gastric ulcer; indeed, it is one form of ulceration, that is to say that there are symptoms of dyspepsia, with pain, vomiting, and hæmatemesis. The hæmatemesis, however, is rarely as profuse as may occur in gastric ulcer. But whereas gastric ulcer occurs in the young, cancer is most common in later life, and again, gastric ulcer very frequently ends in recovery, but in cancer the condition of the patient always gets progressively worse. In some cases cancer causes a tumour, which may be felt through the abdominal wall. If the cancer is situated at the pyloric end of the stomach it leads to obstruction. Obstruction at the pylorus has been mentioned in a previous paper as a cause of vomiting, and the character of the vomit was also described. There is another consequence of the obstruction, namely, dilatation of the stomach, which may be evident by an increased prominence of the abdomen over the area occupied by the stomach.

In cases of dilated stomach, relief may often be obtained by washing out the stomach either by means of a stomach-pump, or, if that is not at hand, a piece of indiarubber tubing with a glass funnel at the end, will answer the purpose well.

The funnel is first elevated, and a certain amount of fluid is poured through it into the stomach; the funnel is then depressed below the level of the stomach, and the fluid flows out. The fluid, of course, must be warm, and may be plain water or water containing some mild antiseptic. Otherwise the treatment of cancer is similar to that of gastric ulcer, except that morphia may be needed for the relief of the pain.

### An Annual Problem.

How is it that in so many reports of Christmas festivities in hospitals we read that presents were provided for the patients by the sisters and nurses? If a Christmas tree is given by an outsider the gifts hung on it are frequently contributed by the staff, and it does not need much acuteness to see that the shrub itself is a very small part of the expense incurred. Do the guests who admire and talk of the joys of Christmas within the wards realise that the women who give their time, their talents, and sometimes their health to the service of "God's poor" are obliged at Christmas to give also of their substance? Of course, some of these workers have private means, and can, therefore, freely indulge themselves in that greatest of luxuries—"giving." But a great many women have no income beyond their salaries, paid quarterly, and we shrewdly suspect that during the three months after Christmas many a generous unselfish nurse has some difficulty in "making both ends meet" in her own private receipts and expenditure account.

It is probably only another example of the evil wrought by want of thought, for there is no lack of heart in the prosperous members of committees who make a smiling progress round the decorated wards and graciously commend the handsome tea-tables and Christmas trees, adorned and furnished by the not over-paid workers over whom they are themselves morally bound to exercise a just and thoughtful supervision.

### Christmas in New York.

As Spent by a Nurse.

Christmas Day is observed chiefly as a religious festival in New York, even the feasting being on a strictly moderate scale. There is no holly, and mistletoe is sold at ten cents per tiny spray, just a mere sprig for a button-hole.

English visitors, or residents with English friends or connections, eat turkey, and add to it excellent cranberry sauce; indeed, bread sauce is seldom seen in the U.S.A. Highly spiced mince pies are made about the size of ordinary dinner plates. Sometimes we see an English plum pudding, and at hotels it is invariably served up with a piece of cheese lying on it.

Our first Christmas Day out here was spent in a children's infirmary, and the little patients had a capital tree.

Christmas festival is shadowed over and greatly absorbed into the fête annually held in the last week of November. There are then free dinners given to the needy, and many geese and mince-pies are consumed, and numerous other delicacies, too. The children are specially regaled with tea and cake and receive many presents.

Of course, the Christmas trees in hospitals are much like those in fashion in England, just prepared and used for the one occasion. In private houses in New York, however, the regular German fashion prevails of having a tree placed in a window weeks beforehand. The ornaments are kept from year to year, and the number is steadily increased. In some of the American hospitals presents are now given specially at Christmastide, and this is done with lavish generosity, in fact, on that large scale which characterises the deeds of our good friends in the United States of America.

A Ward in New York Hospital.

The second anniversary we spent with our family, all dining at a big hotel.

The third one we were private nursing. I myself was in attendance on a lady who was unable to leave her bed, so she and I passed a very quiet day together, for she sent off her good husband to dine with his own relations.

Private houses are generally decorated with wreaths of evergreens and everlasting berries, and hospitals are similarly dressed up, and the sick children have Christmas trees lighted up within a few days of the festival. Many presents, too, are exchanged, but the universal rejoicings and merry-makings, as well as the distribution of charities, take place on Thanksgiving Day.

Great is the feasting then, and, in fact, in New York, the

### Christmas at the New York Hospital, New York City.

A day or two before Christmas quantities of Christmas greens are distributed in the wards, and each head nurse or nurse-in-charge decorates her special ward according to her own taste. And there is often a pleasing variety of arrangement. Early in the morning begins the expression of the day's true meaning, for the nurses, before going on duty, pass through the corridors of the hospital singing appropriate carols. Then the dinner is a more elaborate repast than the usual one, and everything else seems to have a more festive appearance than usual.

Late in the afternoon the female patients have a decorated Christmas tree placed between their two wards, and each woman is the recipient of an appropriate gift.

Some of the nurses have a half holiday, but all prefer to be back at the hospital by evening, when the children's ward is the delight of all present. It is decorated with branches of holly, wreaths of evergreens, sprays of mistletoe, arranged with most artistic effect. In the centre of the ward a large tree is resplendent with coloured electric lights, the tree revolves slowly and continuously, and the little ones, propped up in their beds, clap their tiny hands, and smile with pleasure at sight of the beautiful tree, over which is scattered silvery powder, glistening like frost under the electric lights, while balls of cotton suspended from the tree remind the boys of the days when they were out snowballing one another.

Dolls, picture books, and toys of various kinds are distributed among the children, who seem for the time to forget their sufferings. The babies and younger children wear dainty flannel frocks on this occasion over their white gowns —the blondes have them of a delicate blue, while the brunettes have red ones. It is claimed by many visitors that there is no Christmas tree anywhere in New York City so beautiful as the one presented yearly to the New York Hospital by its Governors. R. B. H.

### Christmas in India.

LUCKNOW.

Our Christmas tree at the Lucknow Hospital last year stood in the verandah, which was brightly illuminated, as was also the yard without, and, of course, the wards within. The tree was a magnificent one, and the gifts which covered it were sent out by English friends, and were very numerous and most acceptable. On Christmas Eve there was a grand gathering of Christian workers as well as the patients and servants, and a little service was held in Panjabi. Afterwards the presents were distributed and gave unbounded delight. Apparently the excitement of the whole affair prevented the natives from sleeping, for they disturbed us between three and four a.m. by making a hideous din outside the hospital.

Awakened by the howls and shrieks, we flew to the windows to find it was a band of native instruments, accompanied by a small firework display. Little fire-balls were thrown sharply on the ground and exploded noisily. Of course, we could only return thanks to our coloured friends for this complimentary ovation, and then retire again to our pillows, longing vainly for silence.

Next day there was a great gathering of old patients, the tree being redressed, and great joy displayed at the distribution of gifts.

We had a grand Tamasha (fête) in the wards on Christmas Day. Having managed beforehand to wrap up a number of small presents received from England in separate parcels, we had a "fish pond." Oh, how the women enjoyed fishing the things out. Dolls were as usual in great demand; they are always as much, or even more cherished by adults than by children in India. A grown-up woman receives one with the greatest joy; she will dress it and care for it, and eventually reluctantly hand it on to her child or grandchild. Old ladies of sixty have been known to weep if left out when a distribution of dolls was taking place, and the delight which natives of all ages take in them must be seen to be believed.

The lady doctors at Lucknow are fully-qualified medical women, and there is an excellent record of work done by the Indian Female Normal Society.

### Presentation.

Glasgow Training Home for Nurses.—Miss McAlpine, the honorary Lady-Superintendent was presented with a silver tea service and an illuminated address on the 14th inst., and Miss Jessie McAlpine received her own portrait. The presentation was made by the Hon. Lord Provost Bell in the name of the nurses, 106 in number, belonging to that institution. Sir John Burns, Bart., replied for the Misses McAlpine, and Lord Overton and Mr. J. C. Alston also made speeches. The nurses had an At Home in the evening.

### Christmas in Northern India.

AMRITSAR.

Christmas under a hot sun, and a glowing sky, Christmas decorations of roses, geraniums and sweet briar. A sharp contrast indeed to the cold, foggy, gloomy climate we too often associate with this season in England. Yet the real joy of Christmas-tide is none the less, and many of the time-honoured English customs find their way to foreign lands, for where English men and women are gathered together, Christmas trees, turkeys and plum puddings seem to follow in natural sequence.

At St. Catherine's Hospital, Amritsar, all the good old home customs prevail, and the boxes of presents and useful and pretty things from England are looked for, and eagerly welcomed. The matron has a busy time with preparations and the record of her Christmas week last year left one lost in wonder at the energy exhibited in carrying so much through with such marked success. Christmas Eve was full of work. The morning spent in hospital duties, the afternoon devoted to receiving notes, cards, and gifts of all kinds.

After church on Christmas morning books were given to all who could read, while those who could not were made happy with pretty handkerchiefs. The blind patients were presented with handkerchiefs, and it is specially desired that these have coloured borders.

A feast for the sick folks in the hospital is managed after the morning service, and the rest of the day is spent quietly. Carol singing gives much pleasure to all.

But Christmas Day itself by no means ends the last of the festivities. An entertainment to a goodly party of girls from a neighbouring school, and a physiological magic-lantern exhibition managed by the matron, with a friend's help, was much enjoyed on the next afternoon.

A further and more extensive gathering together of all the Christian women of Amritsar took place on December 27th. There were 140 guests in all. Presents again formed an important part of the programme, which also included games, and a tea beautifully managed by the native servants. Afterwards, a bright and cheerful service, in Hindustani, of course, with addresses by the various missionaries, was held in a tent in the hospital grounds.

And all this time the usual hospital and dispensary work had to be carried steadily on. Most kindly did the busy medical missionary take an active part in all the festivities, and after a hard day's work acted "Father Christmas" in the Public Gardens for the amusement of the European residents' children.

Truly, Christmas is a busy time in other lands than ours, and the efforts so generously and heartily made to carry the Christmas message of peace and joy, love and goodwill, to our brothers and sisters in far-away India, merit our warm and heartfelt sympathy.

### An Unfortunate Accident.

A very sad interruption to the Christmas entertainment at the Royal Surrey County Hospital resulted from the injudicious act of one of the guests. Some spirit was thrown by him on to the dish of snap-dragon, and the flames suddenly mounted up and spread to the clothes of the visitors standing by. Thanks to the prompt action of the staff the fire was speedily extinguished, but not until four of the guests had been considerably burnt. Happily the patients all escaped injury, although they were naturally much alarmed by the deplorable occurrence.

### Notes and Queries.

**Answer.**

*Offensive Breath.*—We never prescribe. Consult your family doctor.

### Christmas Entertainments.

The patients at the Royal Free Hospital in Gray's Inn Road, thoroughly enjoyed the entertainments got up for their pleasure recently by some of the women students. Such patients as were up went into the lecture room, where tableaux, acting, and music formed an excellent programme. Whilst this was in progress other students went round the wards, which were looking particularly bright and pretty, and there devoted themselves to the diversion of the sick men and women confined to bed. Father Christmas made a dignified progress, in which he was accompanied by the diminutive representative of the New Year. A parcel was presented to each patient, containing useful garments as well as oranges, crackers, sweets, &c. Carols were sung in each ward to the accompaniment of three violins, and many voices joined in the Christmas hymns. One little fellow of six, who had joined with great spirit in "Nowell" was rather exercised in his mind by the appearance of a gentleman amongst the guests, and exclaimed, "Why, I b'lieve there's a doctor a coming to see us, too! I don't see why ever he shouldn't if he's a mind to!" The same urchin explained that he knew all about Father Christmas—"He comes down the chimney, yer know!" Another boy was asked if he had ever met Father Christmas before, and rejoined, "Oh yes, I knows her, she's my lady doctor!" On Christmas Day a sumptuous repast replaced the patients ordinary noontide meal, and presents were distributed. Music, singing, and games followed the excellent tea provided in each ward.

Guy's Hospital.—The wards were prettily decorated with evergreens, flags, &c., and an excellent Christmas dinner was served to the patients. Music and singing took place in the evening, and a band of students gave a famous entertainment, which will be repeated during the week until all the wards have been duly visited by the party.

The Seamen's Hospital, Greenwich.—The patients at this hospital come from all quarters of the globe, and we find that 26 nationalities were represented on Christmas Day by the party who partook of seasonable good cheer. The decorations were very tasteful, and a model of an ice-bound ship attracted much attention in one of the wards.

St. Bartholomew's Hospital.—The wards at this fine old hospital were decorated and a Christmas dinner was served to the patients, followed by games, music, &c., in the various wards. A Christmas tree provided for the children was dressed with presents said to be provided by the liberality of the nurses.

Charing Cross Hospital.—Entertainments were given by the students three days last week to the patients at this hospital, and were greatly appreciated. On Christmas Day a tree was provided for the children and a special dinner for all the inmates who also partook of some of the "Queen's Wine." All the wards were prettily decorated, and the patients received useful gifts.

St. Thomas's Hospital.—A faint mist over the river through which barges slowly drift in the wake of fussy steam-tugs; queer shadows from blinking gaslights; muddy roads and pavements, and a ceaseless stream of foot passengers; that is what we see as we cross the wide bridge at Westminster. Arriving on the other side we pass various blocks of buildings, and finally arrive at the front entrances of St. Thomas's Hospital. Through the swinging doors into the great hall and along the seemingly interminable corridor. And what a curious "Sabbath calm" prevails, although "something in the decorating line" in one doorway engrosses the attention of a group of young men, and a ladder, &c. In the nurses' pretty sitting-room trim maids have put the finishing touches to some tasteful decorations, and the bright holly berries shine forth amidst the dark prickly leaves. Christmas, however, is centred in the wards, and is there a cheerfully accepted fact. On all sides are dainty festoons of evergreens, and trails of ivy with gay flags and coloured lanterns. Patients all seeming interested, and nurses, however busy, finding time "to do a little decorating" between their more serious labours; doctors and students assisting, and also suggesting additional adornments. They are watched earnestly by eyes which have seldom, in their owners' joyless lives, seen the study of beauty brought so closely within their ken. On the evening of Christmas Day the Nightingale probationers go through all the wards singing carols, and the Treasurer makes his usual round and speeches. The patients being regaled with plum pudding and roast beef for their dinner, are entertained at tea later on by each ward sister. On Christmas and Boxing Day, the wards are lighted up, and very charming they look, with fine plants and ferns down the centre of the polished floors, and the rows and rows of beds and shining furniture reflecting back the bright lights. Concerts are given in the wards on other days during the week, and altogether Christmas is certainly well observed at St. Thomas's Hospital.

King's College Hospital.—Presents for the patients and Christmas cheer for everybody in the hospital, but very little decorating is now permitted at King's College Hospital, except, and this is an important exception, in the beautiful little chapel, which is made lovely before the Christmas Eve services begin—scarlet geraniums and brilliant poynsettiers and fair white flowers and delicate ferns, tulips in masses growing in boxes which are cunningly hidden in ivy wreaths. Surely never was a hospital chapel more lovingly and fittingly adorned for the festival of Christmas. An excellent little organ has been presented by the nursing staff, and the fine, familiar harmonies, whose echoes penetrate to the sick folk in the wards, are keenly enjoyed by the congregation assembled to enjoy services and sacred concerts. The seats for the nurses and for such patients as can sit up are specially placed to leave a wide space for the invalid couches to stand three or four abreast, and so many a patient comes in who could not otherwise be present.

The London Hospital.—A fine trophy of flags has been loaned to the fine and lofty receiving room, and there are other appropriate decorations too, besides holly and mottoes. Among the latter are deservedly conspicuous Shakespeare's lines—

"How poor are they who have not patience,
What wound did ever heal but by degrees?"

The wards are much less decorated than usual. "It seems a pity, doesn't it, now?" said one of the porters, "and such a many people offered to send in green stuff But the matron, she says so many nurses having had influenza here as well as at other hospitals, she'd sooner they didn't have anything extra to do now they're all a gettin' well and strong. I suppose she's right," concludes the man, with a sigh, "but we've always been A1 in dressing the wards before," If the wards themselves are "less dressed" than usual, visitors seem unconscious of the fact, for the patients are particularly smart—scarlet capes and rugs and snowy sheets and shirts, whilst as for the children they were almost as proud of themselves as their nurses were of the gay looks of their charges. The patients' tea tables were furnished by the sisters after a most appetising fashion, dessert, crackers, &c., being added to the cakes, coffee, and thin bread and butter. A piano organ kept the children dancing (where their legs permitted of the exercise), and the music seemed to please all the small patients. Some of the gentlemen from the Oxford House Mission gave an excellent magic lantern entertainment, and two doctors exhibited another fine lantern. A most amusing little farce and a waxwork entertainment; also a great deal of good music and many spirited songs were given in the wards. It was puzzling for a stranger to believe that those energetic performers who went from ward to ward repeating their jokes and antics with wonderful spirit before each fresh audience of invalids were actually the hard-working "residents" who had still their professional "evening rounds" to accomplish. But certainly stray visitors—and there were a good many of these—might well be forgiven for failing to recognise them disguised in costumes which puzzled even those who knew them well.

Hospital for Sick Children.—The fine hospital in Great Ormond Street was open to visitors on the 27th inst., when Christmas trees and coloured lamps were much in evidence. Some of the wards had had their "treat" on Christmas Day, and perhaps this accounted for the small number of visitors present on the second occasion.

Sussex County Hospital.—The decorations at this hospital were as charming as possible, much time and taste having been devoted to the adornment of the wards with art muslin, evergreens, &c., cotton-wool, Chinese lanterns, and gay ribbons. Each patient received suitable and useful gifts. Long before Christmas Day dawned the Matron, nurses, and wardmaids marched in procession round the wards, each bearing a lighted candle and all singing carols. Patients and nurses were regaled with Christmas fare during the day, and entertainments were held in the wards during the evening.

National Hospital for Diseases of the Heart.—This hospital, situated in a quiet corner of Soho Square, was a festive scene on Saturday evening. The consulting-room was utilised for the Christmas tree, which was surrounded by present and past patients and friends of the hospital. The distribution of presents to the little folks caused much excitement, and the company afterwards moved on to the women's ward, where the meetings of the Linnean Society were held during the late Sir John Banks' tenancy. A liberal programme of music, songs, and recitations were provided, and the entertainment concluded with the singing of some carols by the choir boys of St. Ann's, Soho.

### Our Christmas Competitions.

In addition to the parcels which were acknowledged in The Hospital of December 23rd, we have received a beautiful consignment of useful articles from Madame Monchablon and her staff. Amongst other gifts sent by these kind and regular contributors are six delightful bed jackets for male patients to wear when sitting up in bed. They are of thick swansdown calico with collars and cuffs of Turkey red, and the admirable addition of a conveniently placed pocket cannot fail to be appreciated by any invalid who has experienced the worry of "hunting around" for the handkerchief, which is never in the right place, however carefully tucked under the pillow. The pretty flannelette nightgowns will be of great value to little children whom croup, bronchitis, &c., have rendered specially susceptible to chill. The serviceable women's petticoats, shawls, comforters, socks and stockings are alike welcome. We have sent parcels to the North London Hospital for Consumption, the Metropolitan Hospital, and the Hospital for Incurable Children, Maida Vale, in addition to the institutions mentioned last week.

The following are extracts from some of the letters acknowledging the receipt of these welcome gifts: "We return very grateful thanks for the parcel of clothes, which are most acceptable, especially at this season of the year." "Our best thanks for kind gift of clothing for patients." "We beg to acknowledge, with many thanks, a parcel of warm and most useful clothing. We are very grateful for this help." "Acknowledge most gratefully the receipt of nice warm clothing for patients." "The kind presents will be very useful and give great pleasure." "The rose-coloured flannel garments are charming, and will be most valuable for delicate children." The socks and stockings are highly esteemed, and we have pleasure in congratulating our kindly workers upon the vast amount of pleasure and comfort which their gifts have bestowed on many sick poor in hospital wards.

### For Reading to the Sick.

### THE SEASONS.—TO THE OLD.

We are passing quickly to the end of the year, to the end of the century, to the end of our lives; and when we recall the beginning of each we find they have had similar experiences attending them. There has been the hopeful spring, the glorious noon, the fruitful autumn. And the ending, how is it to be with each? We will take the century first. In the year 1800 men were tiring of private disturbances and foreign wars; famine and troubles at home, the treasure and blood of our bravest squandered on alien shores; with a fresh hundred years before them, they hoped that times would alter for the better, and, indeed, from the peace of 1815 the land had rest 40 years. Then arts and sciences flourished, a thousand things were discovered which, though now necessities, then astonished men by their novelty; music and painting made rapid strides, and the steam engine has been brought to a perfection hardly anticipated. There has been a rich fruition of knowledge in the middle of the century, and yet men who had begun to look upon themselves as more than mortal now exclaim in sadness and discontent, "Vanity of vanities; all is vanity."

Again our present year of grace, 1893, emerged from a dismal winter, and spring burst suddenly upon us with unusual beauty and vigour, melted imperceptibly into a superb summer, which ripened the fruits of the earth by extra heat and sunshine, and caused the autumn to be a time of plenty and content.

Now winter, black and drear, is upon us—with its fallen leaves, its storms of wind and rain, and its dull, foggy days, all speaking of decay. And have not our own lives been much the same as have the years? We have had our youth, full of hope and the wondrous possibilities to be achieved in the long life before us; if we have done something with it, we have neglected more, and we cry out—for our three-score years are nearly gone—"Alas!" Our harvest is past, the summer is ended, and we are not saved!

And yet it is not inevitable that old age should bring sadness and unhappiness, for there is a Balm in Gilead, a Great Physician who is always stooping to heal the wounded and to minister to the sick. "Truly the light is sweet, and a pleasant thing it is for the eyes to behold the sun," yet our Sun of Righteousness never goes down; we may dwell in His fullness for ever. We have, perhaps, been rugged-tempered in our young days, but as years have passed on we have become like the poet's beautiful simile of the "Holly Tree." Our sharp and prickly leaves have turned smoother and brighter as we climb nearer Heaven, till they resemble the laurel of victory. What beauty is there in the grey hairs of old men? The hoary head is a crown of glory when found in the way of righteousness. Blessed are we if we are waiting with our lamps trimmed ready for the bridegroom, who must shortly come. Our weakness and pains remind us that He will not tarry. Let us then gather up the crumbs of wasted opportunities of time, of talents; let us offer them and ourselves through the blood of Jesus, saying, "Lord, now lettest Thou Thy servant depart in peace; when Thou wilt, O Lord, and as Thou wilt, only without sin and shame." With such thoughts in our hearts, such words on our tongues, we may hope to die the death of the righteous, and our last end to be like His.

### Christmas with the Nuns.

By a Worker.

Our Christmas is a very quiet affair, all the patients who are able go home for the festival. Christmas Eve and the preceding days there is a reversal of the usual order of things, for instead of sorrowful faces passing in there is a joyous exodus, so that when those who remain gather round the cheery ward fire at night the parties are very small and homelike. A great deal of interest and care are displayed in the decorations, which are generally very tasteful and effective. The pictures and gas branches in the pretty wards are twined round with evergreens, and the curtain poles are also decorated. The children's cots have their triangular canopies very gorgeously dressed indeed with the aid of gold and silver balls, and it is pleasant to see the pride of each mite in his own particular cot.

The most solemn feature of Christmas Day is the ceremony of bringing the Blessed Sacrament in the early morning to the sick who cannot get to the chapel.

There is the usual amount of good fare. A real Christmas dinner, for those permitted to take it, of turkey and roast beef and plum pudding, and in the evening a festive tea, presided over by the Sister of each ward, with willing help from the nurses. All endeavour to make the evening pass afterwards in true happy Christmas fashion until the early breaking-up time, at which no patient grumbles, although the younger ones linger reluctantly till the very last.

Afterwards the nurses celebrate their Christmas night in the home, the sisters supplying their places in the wards until the night nurses come on. Of course their celebration is much more lively than the patients. After supper music, dancing, games, &c., go on until it is time to break up, and day and night nurses conclude a very busy and happy if not exactly "jolly" Christmas.

### Everybody's Opinion.

[Correspondence on all subjects is invited, but we cannot in any way be responsible for the opinions expressed by our correspondents. No communications can be entertained if the name and address of the correspondent is not given, or unless one side of the paper only be written on.]

### WORKHOUSE INFIRMARIES.

"An Infirmary-Trained Nurse" writes: In reply to the letter by "One Who Knows," I venture to offer a few remarks on some very sweeping statements upon the "officers" in workhouse infirmaries, and I have been myself an "officer" in one of the largest and best of the London workhouse infirmaries for nearly four years. I know something of the management. "One Who Knows" complains of the insolence and general tone of the porters, but as probationer, staff nurse, and sister, I never met with rudeness or insolence from one of them. Had such been the case, an appeal to the medical superintendent or steward would at once have prevented a recurrence of the annoyance. As regards the cook, though there was certainly room for improvement, I never found her anything but civil to the matron, the undoubted head of the female staff. As regards the objections to chronic cases, from a nursing point of view, I fail to see how "One Who Knows," calling herself a nurse, can find no "special interest" in a "trying chronic case." "Trying" to patience and temper they certainly are, but I will never admit that a good nurse will weary of her work because it is wanting in the interest and excitement which belong to acute cases. The assertion of "One Who Knows" that "a good class of women will not be easily obtained until some changes are made" hardly accords with the fact that at St. Marylebone Infirmary, which is looked upon as the best of the kind both as an infirmary and a training school for nurses, the matron has no difficulty in obtaining any number of suitable women to train as probationers. In fact there are always more applications than vacancies, and as to the class of workers there, they are acknowledged to be good women, content to work hard, and certainly the majority of them "active minded" enough not to weary even of chronic cases.

### Causerie from New England.

### BOSTON.

In this year of "hard times" the hospitals which do not have municipal appropriations or permanent, well-endowed funds, have found it difficult "to make both ends meet." This is particularly true of the hospitals in the smaller cities, the outgrowths of local enthusiasm. But the present dilemma is a valuable test of the public spirit, in which, as a rule, American towns are not lacking.

### A Cottage Hospital.

The Newton Cottage Hospital needs 20,000 dols. for the current year, and a contribution taken last Sunday in the churches aggregated over 8,000 dols. The balance will be raised by a grand co-operative effort in a bazaar or some form of entertainment. The addition of a new plant for heating and a new dining-room has increased the year's expenses about 5,000 dols.

### Quincy City Hospital.

Newton's neighbour, the City of Quincy, finds itself in a still worse condition financially. By January 1st, 1894, the hospital will face a deficit of 5,000 dols. It is not a municipal institution, although it bears the name of Quincy City Hospital. The endowment fund now amounts to only 35,000 dols., invested in real estate and city bonds, and will not be sufficient to meet the expenses for three or four years. Quincy has had a sad object lesson in the utility of a hospital, for almost before the little building was fully equipped a railway accident in the vicinity filled every bed with the injured and dying. The last week in November was known as "Hospital Week," and hundreds of citizens devoted themselves to raising funds. There was a contribution taken on the Sunday to begin with in all the churches. A committee organized in each ward that every family should be visited and solicited to give something, even though it be "the widow's mite." This is largely the work of Quincy ladies.

### A Board of Visitors.

Boston has a new departure in a board of visitors—three men and two women, appointed by the mayor and confirmed by the city government. They serve, without pay, in the interests of humanity, and their duty is to inspect the public institutions of the municipality devoted to the care of paupers and the insane, and also the various reformatories. But this does not include the Boston City Hospital, which is an incorporated body with a special board of trustees. A difference of opinion among those competent to judge of the advisability of this measure will cause the results to be closely noted. There is a popular belief that abuses exist, and the new plan of inspection was the outgrowth of considerable newspaper agitation.

### A Scarlet Fever Epidemic.

Boston has an epidemic of scarlet fever, and the City Hospital has provided special accommodation to meet the unusual demand. To refuse to admit these children, even though the regular scarlet fever ward was crowded, and have them return to their homes where they could not be properly isolated, was a menace to public health.

### Prompt Measures.

In a retired spot not far from the hospital stands an unused emergency hospital, built last year when the Board of Health feared an invasion of cholera. This has been borrowed until the new buildings of the City Hospital designed for contagious diseases shall be completed. The temporary ward thus secured has forty-eight beds. For several weeks the hospital daily records have shown between seventy and eighty cases of scarlet fever.

### St. Katharine's Hospital.

In connection with recent Press comments on the large proportion of the endowment of St. Katharine's Hospital hitherto swallowed up in salaries, we have much pleasure in giving in full " the recommendations " submitted to the County Council, as reported in the *Standard* of December 20th :—

Mr. John Hutton (chairman) presided at the weekly meeting of the County Council, held yesterday, at the County Hall, Spring Gardens.

A lengthy report was submitted by the Corporate Property, Charities, and Endowments Committee on the question of St. Katharine's Hospital, Regent's Park. A history of the hospital was given from the time it was founded and endowed by Queen Matilda in 1148, on the east side of the Tower of London, to accommodate one master, three brother chaplains, three sisters, and six poor scholars, till the year 1878, when new rules were drawn up by which the hospital was at present regulated. With a view to the Council availing itself of the Lord Chancellor's consent to receive suggestions, the committee had had under consideration various matters as suitable for the purpose of modifying the existing scheme, and they recommended "(*a*)That the salaries of the chapter and the officers should be revised and the constitution of the chapter so modernised that all future appointments should be made with a view to there being but one clergyman retained who should hold the office of master, and for the other clergymen duly qualified medical persons should be substituted, whose duties would be to supervise, instruct, and assist the nurses connected with the hospital and institute. (*b*) That additional nursing homes or institutions, for sending nurses to the homes of the poor in times of sickness and in maternity cases, should be established under centres in the north, south, east, and west, the existing hospital of St. Katharine and the Queen Victoria's Jubilee Institute for Nurses being the head establishments. A central home in East London should be first opened, to be followed by those in the other districts as funds admit. (*c*) That the sisters, as far as practicable, should be the superintendents of the various nursing homes and institutions, including the home for aged or invalided nurses. (*d*) That the Queen Victoria's Jubilee Institute for Nurses should at once participate in the surplus income available for charity. (*e*) That one or more of the existing houses attached to the hospital should, as they become vacant, be set apart as additional homes for aged and invalided nurses. (*f*) That the existing bedesmen and bedeswomen should have, if capable, active duties in connection with the charity and the nursing homes, and that no new appointments should be made. (*g*) That the school should be abolished, and that the moneys expended in clothing, &c., should be utilised in providing scholarships for boys and girls from public elementary schools in the county. (*h*) That, so far as the funds admit, provision should be made for pensions to Queen's nurses and St. Katharine's nurses who from age or bodily infirmity contracted in the performance of their duties are unable longer to act as visiting nurses."

Dr. Collins said he thought the recommendations required a great deal of consideration, although he gave the committee every credit for the report they had submitted. He, however, would move that the report be referred back for further consideration.

Mr. Leon seconded the amendment, which was lost.

After further discussion, the recommendations were agreed to, with one or two slight alterations.

### The Book World for Women and Nurses.

[We invite Correspondence, Criticism, Enquiries, and Notes on Books likely to interest Women and Nurses. Address, Editor, The Hospital (Nurses' Book World), 428, Strand, W.C.]

The English public owes a debt of gratitude to Messrs. L. Raven Hill and Arnold Golsworthy for the excellence of their new "humorous and artistic monthly," entitled The Butterfly. Amongst the plethora of serials issued during the past year this magazine deserves especial notice, more especially perhaps in reference to the current number. The reading throughout is excellent. "Touch and Go," a short story by Beatrice Chambers, if a trifle sad, has a ring of reality about it, and is terse and suggestive. This is followed by more humorously-inclined articles, amongst which an extract from "the child's political manual" is witty and certainly "up to date." The illustrations are perfect reproductions from the pencils of well-known artists, conspicuously to the fore being those of L. Raven Hill and Maurice Greiffenhague.

---

A whole encyclopœdia of useful knowledge is contained in "The Outdoor World, or Young Collector's Handbook," by Mr. Furneaux. Parents who do not object to the introduction of what the housemaids call "litter" to their orderly abodes, will confer a vast amount of pleasure by placing in their boys' hands such a plain and straightforward guide to the mysteries of collecting. After all, boys are certain to accumulate creatures and to enlist the aid of their sisters in the operation, so it may just as well be done systematically and with measures of precaution to ensure the rest of the family against coming across molluscous and unpleasing specimens where least expected. Mr. Furneaux's best articles are on the formation of the fresh-water aquarium, and on insects and insect-hunting; but he has also some excellent chapters on animal life at the seashore which, without being exhaustive, are full of well-arranged information. Some slighter chapters on the vegetable world, and a few words on fossil-hunting help to make up an introduction to science which is all the more likely to be helpful from the fact that Mr. Furneaux only aims at inducing his readers "to make a start." The innumerable illustrations are very delicately drawn, and the plates reproduce the colouring of flower and insect with marvellous accuracy.

The City Diary. (W. H. and L. Collingridge, City Press, 148 and 149, Aldersgate Street.)

We have received a copy of this Diary for 1894, which is as complete as in former years. The pages of the small volume are interleaved with blotting paper. A marker is attached, and useful information for city men and others is provided at the commencement. The price is 1s.

## Figures and Tables

**Figure f1:**